# Digital Citizens or Digital Outcasts? Recent Trend in E-Administration Utilization Inequality Among Old Europeans

**DOI:** 10.1093/geroni/igaf122.2584

**Published:** 2025-12-31

**Authors:** Jose David Lopez Blanco, Marco Albertini

**Affiliations:** University of Bologna, Bologna, Emilia-Romagna, Italy; University of Bologna, Bologna, Emilia-Romagna, Italy

## Abstract

As digital technologies become central to public administration—particularly in the post-COVID-19 push for digital transformation—understanding disparities in e-administration engagement among older adults is increasingly urgent. While digitalization promises efficiency and accessibility, it may also deepen existing inequalities, particularly for older persons facing barriers to technology access and digital literacy. This study examines inequality in e-administration usage among adults aged 65+ across 27 European countries, assessing how the European Union’s digital transformation agenda has shaped the digital divide. Using cross-national survey data from 2016 to 2024, we construct an index of the probability of engagement in e-administration, conditioned on key demographic and socioeconomic factors. This index, derived from a probit model, generates predicted probabilities of e-administration usage while accounting for individual characteristics over time. Our analysis explores how digital inequality has evolved, identifying the administrative levels where disparities persist and the role of demographic and social stratification. Results show that while overall disparities in digital engagement have declined, age remains a key factor, with older individuals facing persistent barriers even after adjusting for socioeconomic differences. Additionally, regional and national policies shape e-administration adoption, with digitalization efforts either mitigating or exacerbating exclusion. These findings highlight the need for inclusive digital strategies beyond infrastructure investment, actively supporting older persons through targeted training and user-friendly e-governance systems. As European governments accelerate digital transformation, ensuring equitable access to e-administration is essential to prevent the digital marginalization of aging populations.

